# Progress in Surgery

**Published:** 1896-09-26

**Authors:** 


### PROGRESS IN SURGERY.

### GENITO-URINARY SURGERY.

**Ligation of the Vasa Deferentia for Prostatic Hypertrophy.**—In the "Progress in Surgery," in THE HOSPITAL for June 13th, 1896, p. 177, and November 16th, 1895, p. 117, reference was made to experiments which proved that division of the vasa deferentia produced atrophy of the prostate, though the testes did not always atrophy, and attention was drawn to cases in which the symptoms of prostatic hypertrophy had been relieved by such an operation. Isnardi has now operated on twelve cases[1] of prostatic obstruction by excision of a portion of the vas deferens, and he considers the results very gratifying, but the details of the cases are not full enough to establish the successful nature of the operation, at any rate, as compared with castration. The author considers atrophy of the testes to be an essential condition of success, and is not content with simple division or even with resection of a portion of the vas, but after resection he fixes the end of the lower stump in the lower angle of the wound, allowing it to project about one-fifth of an inch from the skin surface, so as to ensure closure in scar production. He found the testicles did not alter so much in size as in density, and nodules formed in the epididymis. Tilden Brown showed[2] the patient, for the relief of whose symptoms he had ligatured the vasa deferentia, at the New York Surgical Society. Before the operation there was complete retention, though no difficulty was experienced in withdrawing the urine by catheter; a little pus was present in the urine, and the prostate was the size of a billiard ball. Each vas was ligated in two places, but was not divided. At the end of seven days he began to pass the urine by drops to the amount of two drachms, and at the time of his discharge, a month after the operation, and on subsequent examinations, only a few ounces of residual urine remained. The pus disappeared from the urine, and the prostate diminished to the size of a hen's egg, but without any atrophy of the testes. Tilden Brown refers to ten cases operated on by Helferich with very satisfactory results, the patients being able to dispense with the use of the catheter. Guyon[3] contrasts the effects on the prostate of bilateral section of the vasa deferentia with that of castration. Three months after division of the vasa deferentia the trophy of the prostate was much less than after castration, though he thinks in time the result may be the same. His conclusions are based on experiments, not on clinical work. Dumstrey[4] records a case of resection of the vasa deferentia for prostatic hypertrophy with retention and pyuria. The retention disappeared, and the prostate atrophied to half its former size, but the physical strength and mental condition of the patient became impaired. Brashier[5] records a successful case of ligation of both vasa deferentia. The testes, although they got smaller for a time, did not eventually atrophy.

**The Treatment of Prostatic Hypertrophy by Methods other than Division of the Vasa Deferentia.**—Dr. Meyer, of New York,[6] records three cases of prostatic hypertrophy treated by ligation of both internal iliac arteries, or Biers operation. One patient was only partially improved, one died of unknown cause seven days after the operation, and the third was not improved at all. In the last case only one internal iliac artery was tied, the attempt to ligate the other having failed through the extreme hardness of the arterial coat. It is an interesting fact that the half of the prostate supplied by the ligated artery did atrophy considerably. Meyer considers that as the two internal iliac arteries alone supply the gland, the reduction in size after their ligation ought to be permanent, as he thinks that their communication with other arteries through the capillary system will not allow of sufficient re-establishment of the circulation to cause recurrent enlargement of the gland. The author is in favour of the extra-peritoneal method of ligature, even though two incisions are required, as he thinks that intra-peritoneal operation in these old and very often stout persons, with possibly fatty degeneration of the heart, would not be well borne. Dr. Pilcher, of Brooklyn, thinks the operation one attended by considerable danger, and considers that as with prostatectomy it should be thrown aside in favour of some less dangerous procedure. Six cases of prostatic hypertrophy treated by castration have been reported to the New York Surgical Society by Dr. Pilcher.[7] Dr. Pilcher finds the statistics of prostatectomy give a mortaliy of 15 per cent., but in his own experience the immediate danger of this operation has been much greater, the fatal results being due, he believes, to renal complications. The dangers shown to attend both intravesical prostatectomy, or even continuous suprapubic drainage, seem to him a strong reason why we should resort to castration. In all his six cases of castration there was permanent improvement in the urinary symptoms, and in most of them the general health was also improved. In each case there was atrophy of the prostate and removal of the hindrance to normal micturition. In all the cases the power of voluntary micturition had been lost, but was regained; the amount which the patients were able to pass varied in the different cases. Only in one case did mental symptoms develop, and in this case suppuration and sloughing were present in the wound. Dr. Pilcher considers that the remote possibility of the development of mania after castration, while it should not be ignored, cannot be given much weight as a contra-indication to the operation in the presence of the marked obstructive symptoms for which the castration is undertaken. In a case in which the fibrous element in the prostate predominated, although wasting of the gland was very slow at first, nevertheless, in twelve months a very marked atrophy of the whole organ had taken place. Dr. Briddon[8] records a case of castration for prostatic hypertrophy, in which mental disturbance followed the operation. Bruns[9] has collected the records of 148 cases of castration for prostatic hypertrophy. In 83 per cent. decided wasting of the prostate followed. Although the author thinks that the restoration of the function of the bladder is less certain than atrophy of the gland, he believes we may expect decided relief in a large proportion of cases, and complete cures in a few. In cases of dysuria without retention the frequency and urgency of micturition is diminished or removed, and incontinence may quite subside. If retention has been present for only a few weeks the catheter can generally, he finds, be dispensed with, and this often happens even after months or years of catheterisation. Vesical catarrh, as a rule, disappears. Cases in which nervous depression and mental apathy have followed castration, he finds, have been recorded, and this the author regards as a reason for trying ligation of the vas deferens, the results of which, he thinks, have been encouraging, though he considers that further experience is required before any conclusive estimate can be formed as to its value. Dr. Kelsey, of New York, records[10] a case of castration for prostatic hypertrophy in which microscopic examination of the prostate five and a half weeks after the operation failed to show any signs of atrophy. Before the operation he had been unable to pass urine except by catheter for several months, and the power did not return. Two other cases where castration was performed are reported, one by Mr. Charlton, of Salisbury,[11] and the other by Mr. Farrant, of Taunton.[12] In the former some mental disturbance, and in the second temporary insanity followed the operation. The beneficial result with regard to the urinary symptoms was marked in both. A new method of treating prostatic obstruction has been advocated by Negretto,[13] which consists in making multiple punctures into the prostate from the rectum with a Paquelin's thermo-cautery. There was marked atrophy of the prostate and relief to the symptoms of obstruction, and the author was strongly in favour of a further trial of this method. The rectum was dilated and packed with iodoform gauze above the region of the prostate, and the gauze plug left in the bowel for several days. A case of prostatic retention treated by the application of the galvanic current to the bladder by means of one pole placed in the rectum and the other at the end of a catheter in the deep part of the urethra, is recorded by Minervini.[14] Before treatment the patient could not pass a drop of urine spontaneously, but after thirty applications he could dispense with a catheter. A sense of burning and some tenesmus occurred when the current was changed.

**Tuberculous Disease of the Bladder.** — Mr. Morton has collected[15] the records of cases of tuberculous disease of the bladder treated by suprapubic cystotomy, and publishes a case of his own in which a large area of ulceration completely healed after scraping, and applying iodoform to the ulcers through the suprapubic wound, together with prolonged drainage of the bladder. One kidney was, however, the seat of very extensive tuberculous disease, and the other of calculi, and from the kidney mischief the patient died. Attention is called to the value of Mr. Cathcart's apparatus for drainage of the bladder in such cases. Reference is made to the opinion of Mr. Watson Cheyne and Dr. Pilcher (of Brooklyn) that drainage is much more important than the direct treatment of the ulcers. These authors point out that it would be very difficult, if not impossible, to scrape away all the tuberculous disease from the lax bladder wall. Several surgeons have supplemented the scraping by the use of the cautery, and Mr. Battle employed a solution of chloride of zinc as the surface of ulceration was too extensive to cauterize. Mr. Morton refers to the fact that tuberculous disease of the bladder is often only a part of general genito-urinary infection or may lead to it, but points out that some of the published cases show that sometimes the disease is limited to the bladder, or, at any rate, the patients recover without symptoms of renal or genital tuberculosis. Even if there is evidence of tubercle elsewhere, it has been suggested by Battle that the operation should be performed as a palliative measure. In one of Dr. Pilcher's cases it was quite successful, although tubercle was present in one lung and the epididymis on both sides, and in one of Dr. Bell's cases, in which a tuberculous testis was removed it was successful, though the sinus did not quite close. Sometimes the sinus left by suprapubic drainage in cases of tuberculous disease of the bladder becomes infected by tubercle. A case of this kind has lately occurred at St. Bartholomew's Hospital.[16] Reynolds records[17] some cases of vesical tuberculosis diagnosed and treated by exploration of the bladder by the method of Kelly, which consists in dilating the urethra in the female and exploring the interior of the bladder by light reflected from a mirror, a method of bladder exploration introduced for catheterisation of the ureters. The author admits the difficulty in determining the presence of tubercle by this method, when it is situated close to the orifice of the urethra or the ureters, but claims that the relief of the symptoms, after the application of nitrate of silver to the ulcers, was rapid and marked. Tubercle bacilli were discovered in some of the cases.

[1] Therap. Woch., No. 21, 1896, and Therapeutic Gazette. April 15, 1896. [2] Annals of Surgery, June, 1896, p. 737. [3] American Journal of Med. Sciences, April, 1896. [4] Centralbl. f. Clin., No. 18, 1896, and Epit. B. M. Journal, May 16, 1896. [5] Bristol Med. Chirurg. Journal, June, 1896, p. 150. [6] Annals of Surgery, June, 1896, p. 705. [7] Ibid, p. 687. [8] Ibid, p. 741. [9] Mitteilungen ans den Greuzgelieten der Medizin und. Chirurg. Bd. i. Hft. i., and Epit. B. Med. Journal, April 18, 1896. [10] New York Med. Record, May 23, 1896. [11] Lancet, April 25, 1896. [12] Brit. Med. Journal, May 16, 1896. [13] Bif. Med., Jan. 20, 1896, and Epitome B. Med. J., April 11, 1896. [14] Ibid., April 11, 1896, and Epitome B. Med. J., May 16, 1896 [15] Clinical Journal, July 1, 1896, p. 157. [16] Ibid., May 13, 1896. [17] American Medico-Surgical Bulletin, April 4, 1896.

